# Methodology and application of multiplex PCR-dipstick DNA chromatography for the detection of eight respiratory bacterial pathogens

**DOI:** 10.3389/fcimb.2025.1558612

**Published:** 2025-05-27

**Authors:** Liuyang Hu, Xiuri Wang, Qiong Li

**Affiliations:** ^1^ Department of Laboratory Medicine, The People’s Hospital of Guangxi Zhuang Autonomous Region, Guangxi Academy of Medical Sciences, Nanning, China; ^2^ Department of Gastroenterology, The People’s Hospital of Guangxi Zhuang Autonomous Region, Guangxi Academy of Medical Sciences, Nanning, China; ^3^ Department of Microbiology, Guangzhou Baochuang Biotechnology Co., Ltd., Guangzhou, China

**Keywords:** multiplex PCR-dipstick DNA chromatography assay, lower respiratory tract infection, eight respiratory bacterial pathogens, rapid detection, multiple detection

## Abstract

**Background:**

Community-acquired pneumonia is primarily caused by *Acinetobacter baumannii, Pseudomonas aeruginosa, Klebsiella pneumoniae, Streptococcus pneumoniae, Haemophilus influenzae, Staphylococcus aureus, Mycoplasma pneumoniae*, and *Chlamydia pneumoniae*, leading to severe illness and death in developing countries.

**Methods:**

A rapid, straightforward, sensitive, high-throughput, and precise multiplex PCR-dipstick DNA chromatography assay was devised. This innovative technique was specifically engineered for the immediate and efficient detection of the aforementioned eight respiratory pathogens, with particular emphasis on scenarios involving co-infections. Custom-designed specific primers were employed, wherein the 5′ end of the forward primers was tagged with oligonucleotide tags (Tag) and the 5′ end of the reverse primers was conjugated with biotin. A C3 spacer was incorporated to bridge the Tag and the forward primer. Complementary oligonucleotides (cTag) corresponding to each of the eight pathogens were immobilized within the test area of the test strip. Meanwhile, biotin was strategically utilized to create an internal control line at the distal end of the test strip. The biotin moiety at the 5′ end of the reverse primer was engineered to interact with blue latex microspheres coated with streptavidin, thereby triggering a detectable signal. Following the PCR amplification of the target DNA fragments, during the membrane strip chromatography hybridization process, the Tag- and biotin-labeled target DNA engaged in a dual interaction. First, it bound to the blue latex microspheres via streptavidin–biotin binding, and second, it hybridized with the cTag on the membrane strip. This led to the accumulation of captured blue latex microspheres at both the test line and the internal control line, manifesting as visible blue bands. A total of 186 respiratory sputum or bronchoalveolar lavage fluid specimens were collected and analyzed. The multiplex PCR-dipstick DNA chromatography assay was deployed for detection, while traditional bacterial culture was also carried out in parallel for comparative purposes. To rigorously validate the accuracy of the multiplex PCR-dipstick DNA chromatography assay in identifying PCR products, DNA sequencing was performed on all PCR products derived from the clinical samples.

**Results:**

The multiplex PCR-dipstick DNA chromatography assay demonstrated remarkable efficacy, being capable of specifically discriminating among the eight pathogens within a remarkably short timeframe of 40 minutes. The detection limit for individual bacterial species ranged from 10 to 10^2^ CFU/mL. Notably, no cross-reactions were observed among the eight target bacteria, nor with other representative respiratory bacteria, including *Acinetobacter junii*, *Enterobacter cloacae*, *Klebsiella oxytoca*, *Haemophilus parainfluenzae*, *Pseudomonas fluorescens*, *Aeromonas hydrophila*, and *Staphylococcus epidermidis*. The concordance between the results obtained from the multiplex PCR-dipstick DNA chromatography assay and those from DNA sequencing was absolute, with a kappa value of 1.00.

**Conclusion:**

A successful multiplex PCR-dipstick DNA chromatography assay was established for the simultaneous detection of eight respiratory bacterial pathogens and was effectively applied in clinical sample analysis. This indicates that this single-use device has promising potential for analyzing the microbial composition related to respiratory infections and is also suitable for small laboratories and field diagnostics.

## Introduction

Lower respiratory tract infection remains one of the primary causes of hospitalization and death worldwide (Vink et al., 2014; Simpson et al., 2015; Beck et al., 2015; Noviello and Huang, 2019). Diverse etiologies have been linked to acute respiratory infections, with bacteria being significant causative agents of lower respiratory tract infections (Price et al., 2019; Uzoamaka et al., 2017; He et al., 2017; Liu et al., 2018; Carvalhaes et al., 2019). *Acinetobacter baumannii*, *Pseudomonas aeruginosa*, *Haemophilus influenzae*, *Staphylococcus aureus*, *Streptococcus pneumoniae*, *Klebsiella pneumoniae*, *Mycoplasma pneumoniae*, and *Chlamydia pneumoniae* were identified as the responsible pathogens causing pneumonia (He et al., 2017; Khadanga et al., 2014; Yun et al., 2019; Wang et al., 2019). The prompt identification and treatment of bacterial pathogens are crucial, as these pathogens can lead to severe, complicated pneumonia and even mortality. Co-infections of respiratory pathogens occur frequently (Liu et al., 2018; Kotula et al., 2018). There is an urgent need for a rapid, straightforward, sensitive, high-throughput, and accurate diagnostic technique for respiratory pathogens, especially for co-infections involving these agents. However, the traditional microbial culture method is time-consuming and labor-intensive and generally can only identify dominant pathogens.

Multiplex polymerase chain reaction (PCR), owing to its advantages of speed, sensitivity, and specificity, is anticipated to become the most efficacious method for detecting respiratory pathogens. Nevertheless, real-time PCR methods have a narrow diagnostic scope, as they can only detect two or three pathogens concurrently in a single reaction due to the limited recognition capabilities of the instrument (van de Pol et al., 2007; Mangold et al., 2011). With the swift development of molecular biology technology, specific probe hybridization technology (Chen et al., 2020), PCR technology for amplifying specific nucleic acid fragments (Chen et al., 2019), and 16S rRNA sequence analysis (Menon et al., 2017) have also been applied to the detection of pathogenic microorganisms. These methods are sensitive, specific, and rapid, but they all depend on expensive equipment and trained laboratory technicians, which limits their widespread application in small community hospitals and remote areas.

Thanks to its rapidity, sensitivity, and ease of automation, the paper-based microfluidic DNA biosensor has emerged as a promising approach for detecting pathogenic microorganisms in recent years (Özgür et al., 2020; Abdelrasoul et al., 2020; Song et al., 2019). In particular, paper-based microfluidic DNA biosensors used gold nanoparticles (Chen et al., 2020, 2019; Menon et al., 2017; Özgür et al., 2020; Bahavarnia et al., 2020) and color polystyrene beads (Abdelrasoul et al., 2020; Cao et al., 2009) as signal generators to realize the visual detection of target. Accordingly, paper-based microfluidic DNA biosensors can also provide semi-quantitative results for bacterial targets through intensity reading or coupling with other agents like enzymes (Chen et al., 2019), fluorescent dye (Huang et al., 2020; Fadlalla et al., 2020), or radioactive binding compound (Banakar et al., 2022). Given its rapid, compact, sensitive, cost-effective, and disposable nature (Chen et al., 2019; Menon et al., 2017; Özgür et al., 2020; Abdelrasoul et al., 2020; Huang et al., 2020; Ang et al., 2012), an increasing number of paper-based microfluidic DNA biosensors have been designed for point-of-care diagnosis. Moreover, the paper-based microfluidic DNA biosensor does not require complex equipment, reagents, or a power supply, minimizing the demands on laboratory personnel. The test strip DNA biosensor platform can be constructed as needed, enabling the intuitive display of detection results for different pathogens. It holds significant potential for application in the analysis of pathogenic microorganisms and antimicrobial resistance (Tian et al., 2016; Liles et al., 2019; Shanmugakani et al., 2017; Hayashi et al., 2013; Tian et al., 2014).

This study aimed to develop a simple, rapid, and sensitive PCR-dipstick chromatography method and achieve the simultaneous detection of respiratory bacterial co-infections by combining PCR and paper-based microfluidic bioassay.

## Materials and method

### Standard strain

Standard strains of *A. baumannii* (ATCC19606), *P. aeruginosa* (ATCC9027), *K. pneumoniae* (ATCC13883), *S. pneumoniae* (ATCC49619), *H. influenzae* (ATCC49766), *S. aureus* (ATCC6538), *M. pneumoniae* (ATCC15531), and *C. pneumoniae* (ATCC53592) were purchased from the Guangdong Microbiological Culture Collection Center.

### Clinical samples

The study was approved by the medical ethics committee of the People’s Hospital of Guangxi Zhuang Autonomous Region, and all the subjects provided informed consent. A total of 186 specimens of sputum or bronchoalveolar lavage fluid (BALF) were collected from pneumonia patients at the People’s Hospital of Guangxi Zhuang Autonomous Region between December 2023 and December 2024. The collected sputum/BALF samples were characterized by a white blood cell count exceeding 25 and an epithelial cell count of less than 10 under ×10 magnification. These 186 sputum/BALF specimens were evaluated using the PCR-dipstick chromatography assay and were also subjected to culture. To further assess the accuracy of the PCR-dipstick chromatography assay, all PCR products were sent for sequencing.

### Sample preparation

The sample preparation process for the multiplex PCR-dipstick DNA chromatography assay was as follows: equal volumes of 4% sodium hydroxide were added to the sputum/BALF samples, which were then incubated for 10 minutes. Subsequently, the samples were transferred to 1.5-mL centrifuge tubes and centrifuged at 15,000 rpm for 5 minutes, after which the supernatant was discarded. Next, 1 mL of sterile physiological saline was added, and the pellet was resuspended by gentle shaking. The samples were then centrifuged again at 15,000 rpm for 5 minutes, and the supernatant was discarded as completely as possible. Finally, 1 mL of sterile physiological saline was added. The pretreated sputum/BALF samples were stored in tubes at −80°C. Genomic DNA was extracted from the sputum/BALF samples using Magen HiPure Bacterial DNA kits according to the manufacturer’s instructions. For the bacterial culture methods, a sterile swab was used to obtain an appropriate amount of purulent sputum, which was then inoculated onto blood agar, MacConkey agar, and chocolate agar, with sector lines carefully drawn. BALF samples were centrifuged at 13,000 rpm for 5 minutes, and the sediment at the bottom was collected and inoculated onto the same types of agar plates, also with sector lines drawn. All media were incubated at 35°C in an atmosphere containing 5% CO_2_ for 48 hours. After 48 hours, matrix-assisted laser desorption/ionization time-of-flight mass spectrometry (MALDI–TOF MS) was employed to identify the isolated strains. The specific steps were as follows: A single colony was carefully picked and spotted onto a MALDI target plate, dried at room temperature, then covered with 1 µL of α - Cyano - 4 - hydroxycinnamic acid (HCCA) matrix solution, and subsequently dried at room temperature again before being placed into the instrument for analysis. Protein spectra were analyzed using the FlexControl™ software in conjunction with the MBT compass software (Bruker, Billerica, MA, USA) and compared against the Mass Spectrometry Identification (MSI) platform database 2. Identification was confirmed when the MSI score was greater than or equal to 2.0. If multiple identification results were suggested for the same colony, only the result with the highest score was retained.

### Primer and probe design

In multiplex PCR reactions, the presence of multiple pairs of primers can potentially lead to non-specific amplification. In this experiment, 10 pairs of specific primers for each bacterium were found from published literature for preliminary screening. These primers were further tested using the NCBI BLAST to ensure their specificity. Subsequently, the eight bacterial primer sets were incorporated into the multiplex PCR system using a stepwise sequential approach. Real-time PCR with melting curve analysis was performed to verify the specificity of the primers through characteristic melting temperature (Tm) profiles (Hu et al., 2020). Eventually, eight bacterial primers were selected based on their excellent amplification efficiency and the absence of non-specific primer amplicons (Hu et al., 2020; Luo et al., 2021) (**Table 1**). The 5′ terminus of the forward primer was tagged with a segment of oligonucleotides (tag) uniquely linked by C3 spacer (Niwa et al., 2014), in which C3 spacer was used to ensure that the tag remains a single strand during amplification. The 5′ terminus of the reverse primer was labeled with biotin.

**Table 1:** The primers and probes of multiplex PCR-dipstick DNA chromatography assay.

Primer	Gene	Tag	Sequence (5′–3′) (F: Tag-C3-F; R: Biotin-R)	Amplicon size (bp)	Probe
*A. baumannii*-F	*iucD*	cgaagttccgagatggcc	Tag1-C3-GGCTGGACATCATCAACTGC	193	Tag1
*A. baumannii*-R			Biotin-GTCGGCCTGATCTCGTATGA		
*P. aeruginosa*-F	*hpmA*	taactgtaatccgctaggat	Tag2-C3-AGAAGACCGTAAGCCAGACC	244	Tag2
*P. aeruginosa*-R			Biotin-CTACTGGCACCCACTCCTG		
*K. pneumoniae*-F	*yfkN*	tgttctctgaccaatgaatctgc	Tag3-C3-TACACAATCGCCCGTTGAAC	223	Tag3
*K. pneumoniae*-R			Biotin-CCCGGTTAGATCCATGGTGA		
*H. influenzae*-F	*atoE*	gcagattcattggtcagagaaca	Tag4-C3-CTGGTGTTGCGGCTAAAAGT	168	Tag4
*H. influenzae*-R			Biotin-TCATTAACTGGGGCTTCGGT		
*S. pneumoniae*-F	*lytA*	gacacctagatatgattcgaagg	Tag5-C3-GCACACTCAACTGGGAATCC	110	Tag5
*S. pneumoniae*-R			Biotin-ATGCAACCGTTCCCAACAAT		
*S. aureus*-F	*cap5F*	agtactaatacgtgccgattcct	Tag6-C3-AGTCACGTCTCGATCGAACA	175	Tag6
*S. aureus*-R			Biotin-GAAACTTGACCACGATCCGG		
*M. pneumoniae*-F	*mgpA*	tagctaagtggtccataact	Tag7-C3-ACCCAGTCACCGATCTGTTT	140	Tag7
*M. pneumoniae*-R			Biotin-GCTCTGTTCGTTGGTGTCAA		
*C. pneumoniae*-F	*dnaJ*	ctgcgggtatagaagccct	Tag8-X-GCACTTTCATGGGAGCCTTT	92	Tag8
*C. pneumoniae*-R			Biotin-GCGCATTCCAAAAGCTTCAC		
Internal control-F	NM-005157	gctctagccaccaatgaatcta	Tag IC-C3-CAGAGCACAGAGACACCACT	153	Tag IC
Internal control-R			Biotion-GGCGCTCATCTTCATTCAGG		

F, forward primer; R, reverse primer.

Additionally, the inclusion of an internal control (IC) was crucial to ensure the reliability of the results and minimize the impact of sample variability, instrument performance, reagent quality, and operational errors. The IC was a synthetic sequence (CAGAGCACAGAGACACCACTGACGTGCCTGAGATGCCTCACTCCAAGGGCCAGGGAGAGAGCGATCCTCTGGACCATGAGCCTGCCGTGTCTCCATTGCTCCCTCGAAAAGAGCGAGGTCCCCCGGAGGGCGGCCTGAATGAAGATGAGCGCC) (Luo et al., 2021). The homology of the IC with the target genes was extremely low, indicating that it would not trigger cross-reactions. A pair of primers was also designed for the IC (Luo et al., 2021). The primers and the probes (**Table 1**) were synthesized by TBA (Tohoku Bio-Array, TBA, Sendai, Japan).

### Detection principle of multiplex PCR-dipstick DNA chromatography

**Figure 1:**
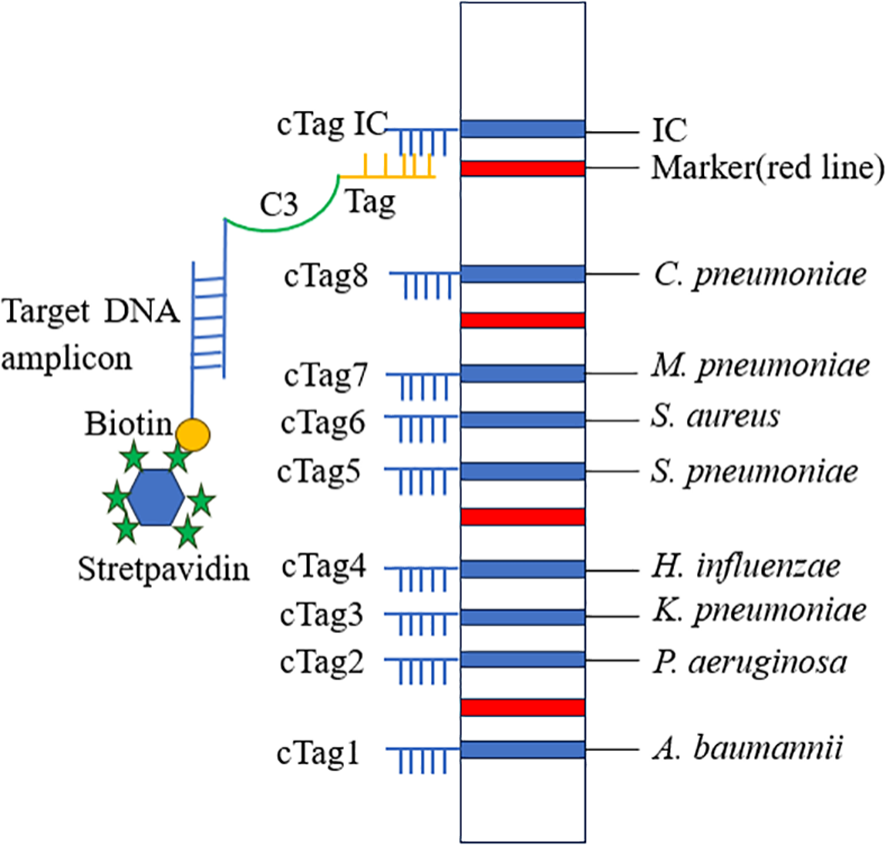
Schematic diagram of PCR-dipstick chromatography assay. Tag, oligonucleotide strand; cTag, complementary oligonucleotide strand.

The dipstick strips produced by Tohoku Bio-Array, Co., contain three main components: a polyvinyl chloride (PVC) membrane that provides stability and adhesion, an absorbent area made of filter paper, and a sample application area made of nitrocellulose (NC) membrane. Complementary oligonucleotides (cTag) are fixed on the NC membrane, forming detection lines for eight pathogens, along with an IC line. Additionally, four red positioning lines are printed on the sample application area. These red lines serve as markers for locating different bacterial detection lines. For each pair of primers of eight bacteria, the 5′ terminus of the forward primer was tagged with eight different oligonucleotides (Tag), and the 5′ terminus of the reverse primer was tagged with biotin. The order of the cTag on the dipstick strip from the downside to the upside is *A. baumannii*, *P. aeruginosa*, *K. pneumoniae*, *H. influenzae*, *S. pneumoniae*, *S. aureus*, *M. pneumoniae*, *C. pneumoniae*, and internal control line. The terminal of the forward primer was connected with a tag by C3 spacer. The insertion of C3 spacer terminates DNA synthesis of Taq DNA polymerase at the insertion site so that tag is left as a single strand in the PCR (Niwa et al., 2014). Target DNA amplicons with an oligonucleotide-tagged terminus and a biotinylated terminus were coupled with latex beads through a streptavidin–biotin interaction and then hybridized with complementary oligonucleotides on the strip. The accumulation of captured latex beads on the test and internal control lines produced blue bands, enabling the judging of the result just by the naked eye (**Figure 1**).

After PCR amplification, 10 μL of PCR product was mixed with 9 μL of eluent (containing detergents, blocking agents, phosphate buffered saline (PBS), and salt solution) and 1 μL of streptavidin-coated blue latex suspension. A dipstick strip was then inserted into the mixture. After 10 minutes, the corresponding test lines and internal control lines were clearly visible as blue lines, indicating that the target bacterium amplicon was positive. It is of utmost importance to note that, regardless of the sample outcome, the IC line must always display a visible blue band. This serves as a critical quality control measure, confirming that the strips are operating correctly and that the assay conditions are suitable for reliable results. If the IC line fails to show a blue band, it signals a potential issue with the strip, the reagents, or the assay procedure, and the results obtained should be considered unreliable.

### Optimization of PCR reaction system and procedure

*S. aureus* was selected as the representative species for optimizing the reaction program and conditions. To ascertain the most suitable reaction system and procedure for the multiplex PCR-dipstick DNA chromatography assay, the standard strains of *S. aureus* (10^5^, 10^4^, and 10^3^ CFU/mL) were amplified by any combination of reaction procedures 1 and 2 and reaction systems 1 and 2. PCR amplification products were analyzed by dipstick chromatography and 3% agarose gel electrophoresis. Reaction system 1 and system 2 were identical in composition, with the exception of the buffers employed. The multiplex PCR was carried out in a total volume of 25 μL. In reaction system 1, the buffer was self-produced 10 × PCR buffer, with a volume of 2.5 μL. In contrast, reaction system 2 utilized 10 × Takara PCR buffer, also at a volume of 2.5 μL. The remaining components of the reaction systems were as follows: 1 μL of dUTPmix (100 mM); 0.4 μL each of the forward and reverse primers (10 μM) specific to the eight pathogens; 0.3 μL of the IC forward primer (2 μM) and 0.3 μL of the IC reverse primer (2 μM); and 1 μL of IC DNA (1 nM), 0.02 μL of UNG, 0.3 μL of Hs-Taq, and 5 μL of DNA template. Deionized water was added to bring the total volume up to 25 μL. Reaction procedure 1 was 95°C for 3 minutes and the following amplification program for a total of 40 cycles: 95°C for 5 s and 60°C for 20 s. Procedure 2 was 95°C for 3 minutes and the following amplification program for a total of 40 cycles: 95°C for 5 s and 60°C for 30 s.

### Sensitivity of multiplex PCR-dipstick chromatography

To evaluate the limit of detection (LOD) of the PCR-dipstick chromatography assay, serial 10-fold dilutions of the DNA from standard strains of *A. baumannii*, *P. aeruginosa*, *K. pneumoniae*, *S. pneumoniae*, *H. influenzae*, *S. aureus*, *M. pneumoniae*, and *C. pneumoniae* were prepared, ranging from 10 to 10^7^ CFU/mL. These diluted samples were then analyzed using the PCR-dipstick DNA chromatography assay to determine the assay’s sensitivity. The lowest concentration at which a visible blue band appeared at the corresponding detection line was deemed the LOD of the PCR-dipstick chromatography assay.

## Results

### Determination of reaction system and procedure

**Figure 2:**
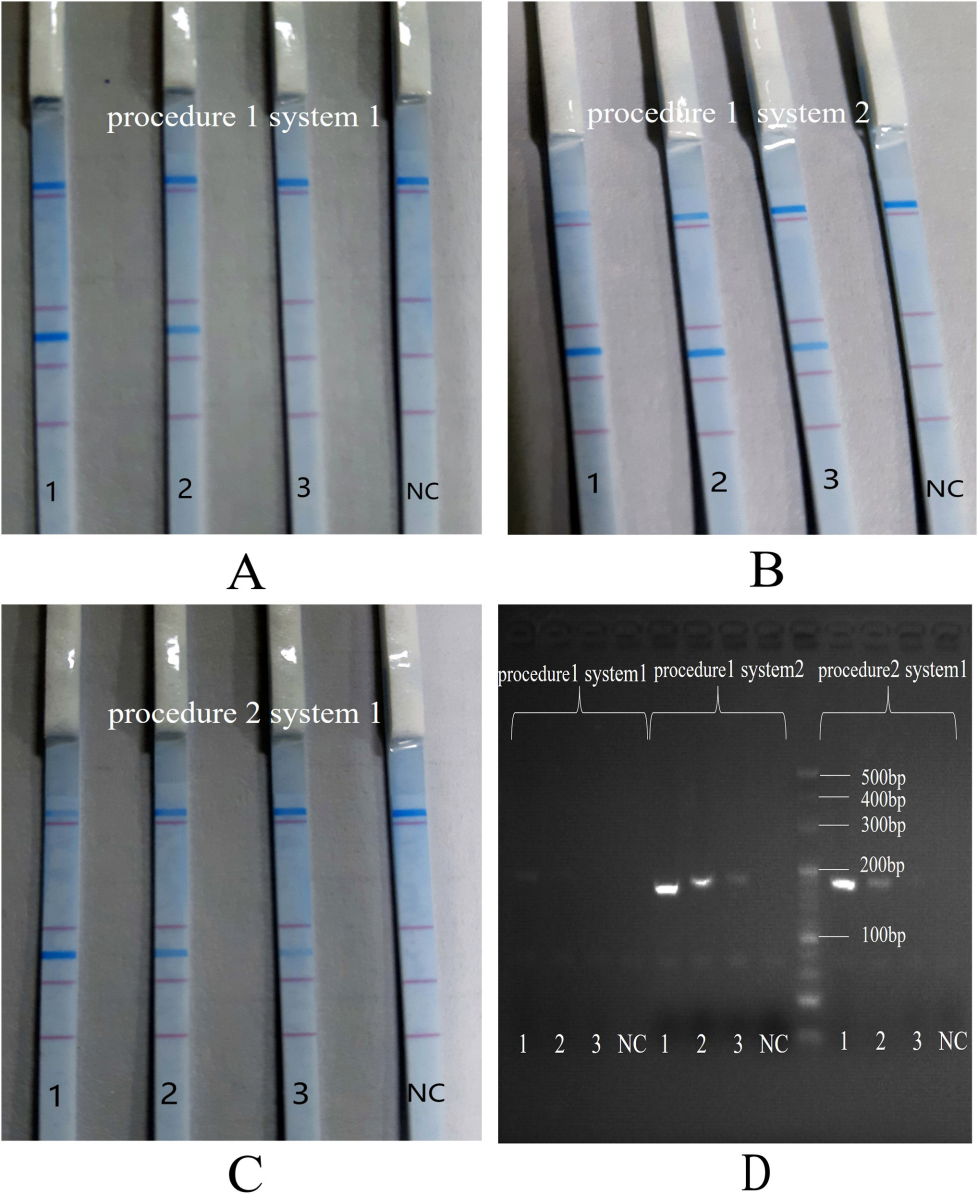
Comparison of PCR procedures and reaction systems. 1: 10^5^ CFU/mL of *S. aureus*; 2: 10^4^ CFU/mL of *S. aureus*; 3: 10^3^ CFU/mL of *S. aureus*; NC, negative control. **(A)**: reaction procedure 1 and reaction system 1; **(B)**: reaction procedure 1 and reaction system 2; **(C)**: reaction procedure 2 and reaction system 1; **(D)**: 3% agarose gel electrophoresis.

The standard strains of *S. aureus* (with concentrations of 10^5^, 10^4^, and 10^3^ CFU/mL) were amplified through all possible combinations of reaction procedures 1 and 2, as well as reaction systems 1 and 2. As illustrated in **Figure 2**, no non-specific bands were detected in any of the three combinations. This demonstrated that the combination of reaction procedure 1 and reaction system 2 yielded the optimal dipstick DNA chromatography performance and the highest sensitivity in 3% agarose gel electrophoresis (as indicated by B and D).

Consequently, the finalized PCR-dipstick chromatography reaction protocol was 95°C for 3 minutes and the following amplification program for a total of 40 cycles: 95°C for 5 s and 60°C for 20 s. The final reaction mixture was formulated to contain 2.5 μL of 10 × Takara PCR buffer, 1 μL of dUTP mix (100 mM), 0.4 μL each of the forward and reverse primers (10 μM) specific for eight pathogens, 0.3 μL each of the IC forward and reverse primers (2 μM), 1 μL of IC DNA (1 nM), 0.02 μL of UNG, 0.3 μL of Hs-Taq, and 5 μL of DNA template, and deionized water was added to adjust the total volume to 25 μL.

### Specificity of PCR-dipstick DNA chromatography

**Figure 3:**
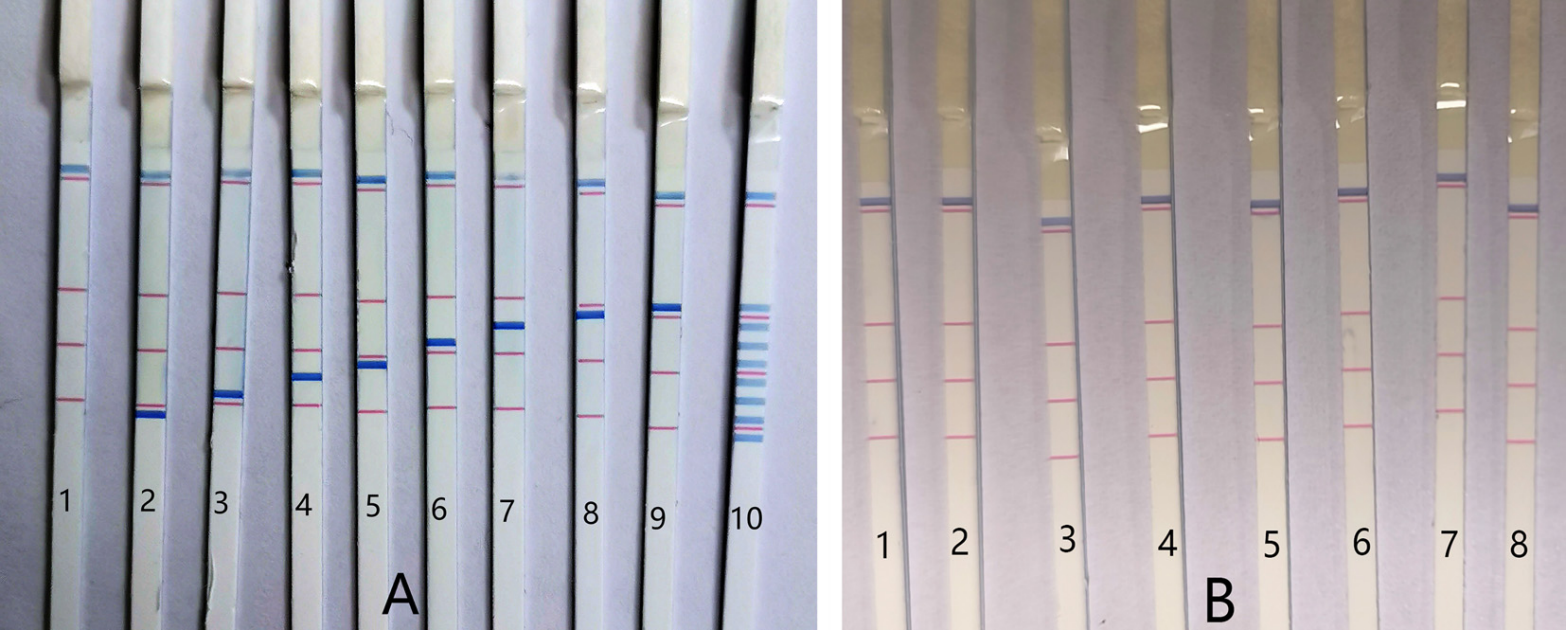
The specificity of PCR-dipstick DNA chromatography. **(A)** 1: negative control; 2: *Acinetobacter baumannii*; 3: *Pseudomonas aeruginosa*; 4: *Klebsiella pneumoniae*; 5: *Haemophilus influenzae*; 6: *Streptococcus pneumoniae*; 7: *Staphylococcus aureus*, 8: *Mycoplasma pneumoniae*; 9: *Chlamydia pneumoniae*; 10: multiplex detection, detect equivalent mixture of eight bacterial standard strains DNA (at a concentration of 10^6^ CFU/mL). **(B)** 1: negative control; 2: *Acinetobacter junii*; 3: *Enterobacter cloacae*; 4: *Klebsiella oxytoca*; 5: *Haemophilus parainfluenzae*; 6: *Pseudomonas fluorescens*; 7: *Aeromonas hydrophila*; 8: *Staphylococcus epidermidis*.

Initially, the specificity of the multiplex PCR-dipstick DNA chromatography assay was assessed to ascertain whether the eight pairs of primers could amplify effectively. The detailed procedure was as follows: each bacterial standard strain (at a concentration of 10^6^ CFU/mL) was detected using the PCR-dipstick DNA chromatography assay to monitor whether blue bands corresponding to the other seven bacteria would appear at their respective detection sites. Sterile purified water served as the negative control. A visible blue band was present at the corresponding test line for the detection of each bacterium, while no non-specific amplification was observed, indicating that there was no cross-reaction among the eight bacteria (**Figure 3A**).

Furthermore, the PCR-dipstick DNA chromatography assay was employed to detect an equivalent mixture of the DNA of eight bacterial standard strains (at a concentration of 10^6^ CFU/mL). It was discovered that each test line corresponded to a specific bacterial standard strain DNA, and there was no false positive in the negative control (**Figure 3A**). These results suggest that this assay is suitable for multiplex analysis. Representative respiratory bacteria, including *Acinetobacter junii*, *Enterobacter cloacae*, *Klebsiella oxytoca*, *Haemophilus parainfluenzae*, *Pseudomonas fluorescens*, *Aeromonas hydrophila*, and *Staphylococcus epidermidis*, were also examined using the PCR-dipstick chromatography assay to verify the specificity of the primers against other bacteria. All the results were negative (**Figure 3B**), further demonstrating that the PCR-dipstick DNA chromatography method possesses excellent specificity.

### Sensitivity of PCR-dipstick DNA chromatography

**Figure 4:**
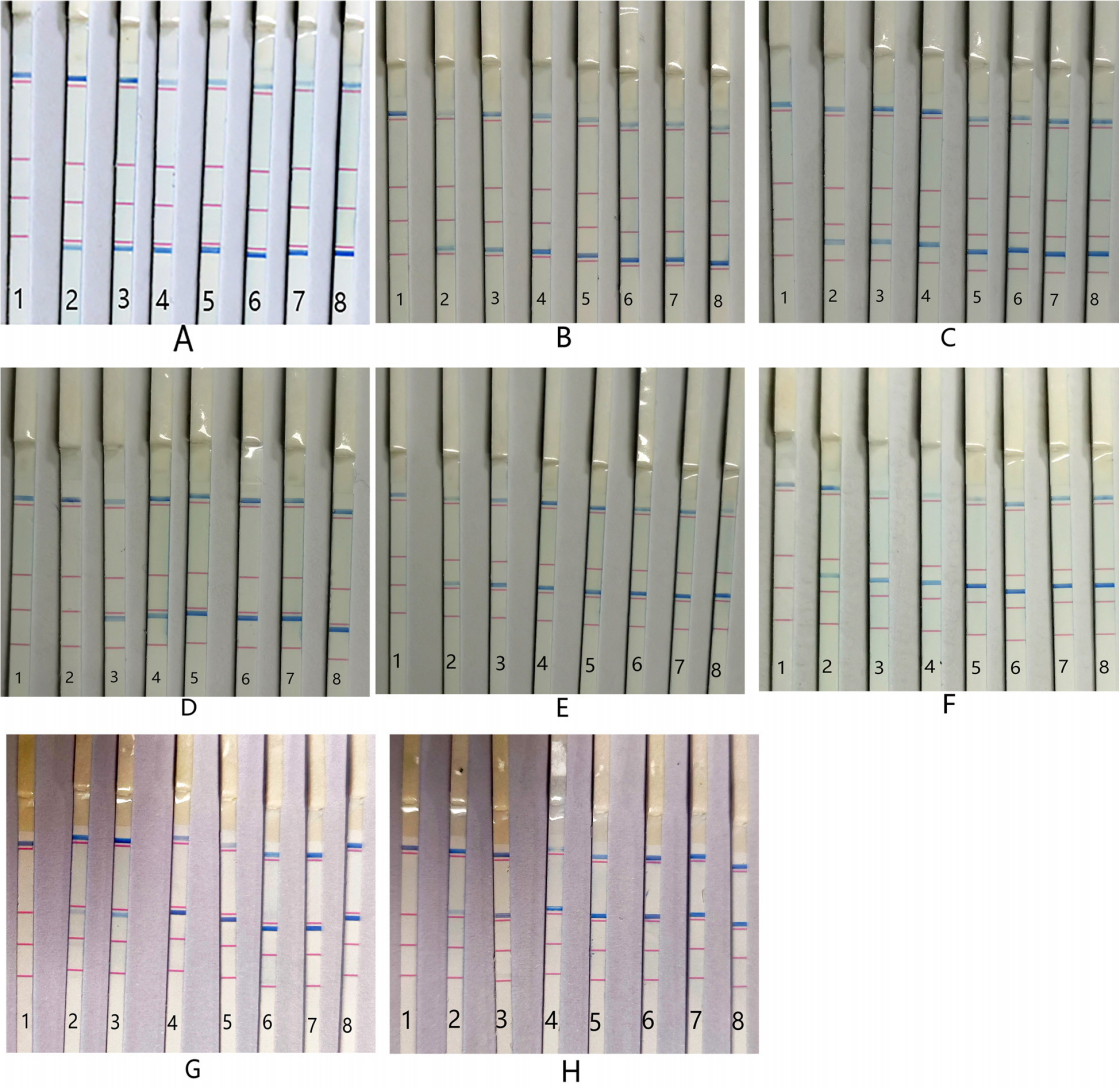
Sensitivity of PCR-dipstick chromatography assay for detection of single bacterium. **(A)** *Acinetobacter baumannii*, **(B)** *Pseudomonas aeruginosa*, **(C)** *Klebsiella pneumoniae*, **(D)** *Haemophilus influenzae*, **(E)** *Streptococcus pneumoniae*, **(F)** *Staphylococcus aureus*, **(G)** *Mycoplasma pneumoniae*, and **(H)** *Chlamydia pneumoniae*. 1: negative control; 2: 10 CFU/mL; 3: 10^2^ CFU/mL; 4: 10^3^ CFU/mL; 5: 10^4^ CFU/mL; 6: 10^5^ CFU/mL; 7: 10^6^ CFU/mL; 8: 10^7^ CFU/mL.

The single-bacterium sensitivity of the PCR-dipstick DNA chromatography assay was determined as follows: for *A. baumannii*, *P. aeruginosa*, *K. pneumoniae*, *S. pneumoniae*, *S. aureus*, *M. pneumoniae*, and *C. pneumoniae*, the detection limit was 10 CFU/mL; for *H. influenzae*, it was 10^2^ CFU/mL (**Figure 4**).

### Clinical sample testing and qualitative analysis

**Figure 5:**
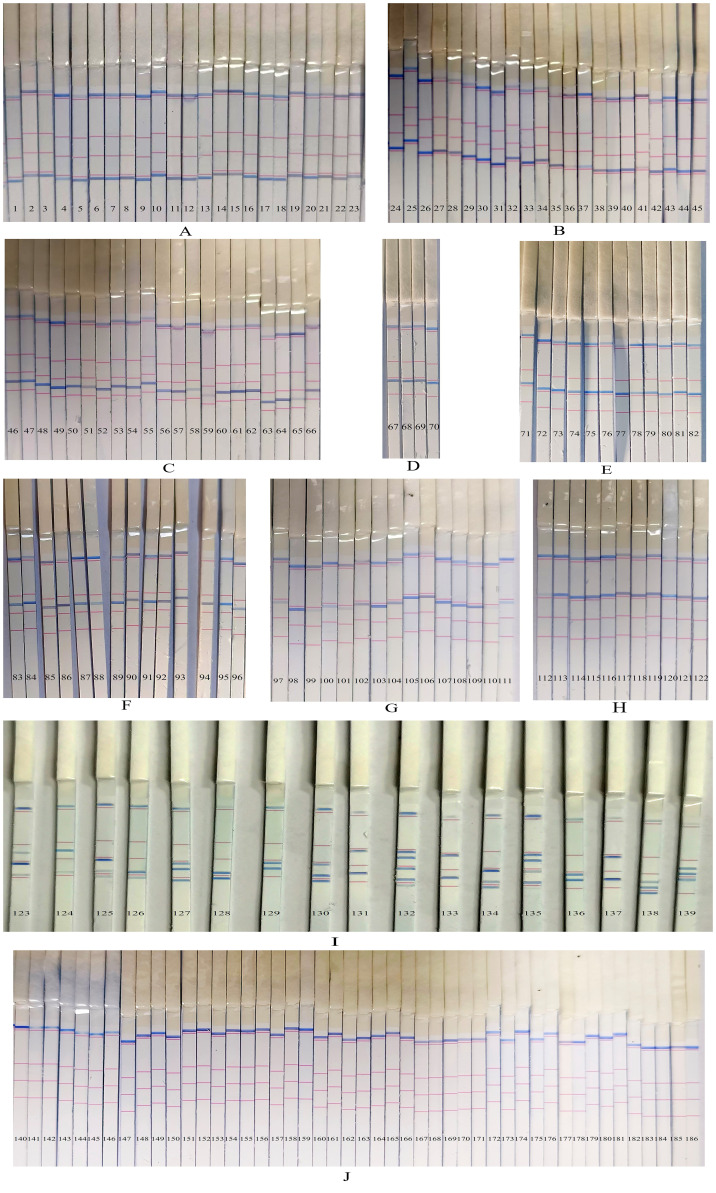
The results of the multiplex PCR-dipstick DNA chromatography method in testing clinical samples. **(A)** *Acinetobacter baumannii* in 23 cases, **(B)** *Pseudomonas aeruginosa* in 22 cases, **(C)** *Klebsiella pneumoniae* in 21 cases, **(D)** *Haemophilus influenzae* in 4 cases, **(E)** *Streptococcus pneumoniae* in 12 cases, **(F)** *Staphylococcus aureus* in 14 cases, **(G)** *Mycoplasma pneumoniae* in 15 cases, **(H)** *Chlamydia pneumoniae* in 11 cases, **(I)** 17 multiple pathogens cases, and **(J)** negative sample in 47 cases.

A total of 186 sputum/BALF samples were analyzed using the PCR-dipstick DNA chromatography assay, and the results were compared with those obtained from the conventional culture method. The outcomes of the multiplex PCR-dipstick DNA chromatography method when testing the 186 clinical samples were as follows: *A. baumannii* was detected in 23 cases (**Figure 5A**), *P. aeruginosa* in 22 cases (**Figure 5B**), *K. pneumoniae* in 21 cases (**Figure 5C**), *H. influenzae* in 4 cases (**Figure 5D**), *S. pneumoniae* in 12 cases (**Figure 5E**), *S. aureus* in 14 cases (**Figure 5F**), *M. pneumoniae* in 15 cases (**Figure 5G**), *C. pneumoniae* in 11 cases (**Figure 5H**), and mixed infections in 17 cases (**Figure 5I**), with 47 cases yielding negative results (**Figure 5J**).

Conventional bacterial culture was also performed on all 186 samples. Among them, 24 samples grew *A. baumannii*. In one sample, the results differed from those of the PCR-dipstick DNA chromatography method, which detected a mixed infection of *A. baumannii*, *P. aeruginosa*, and *K. pneumoniae* (**Figure 5I**—138). This discrepancy between the two methods is due to the fact that the sensitivity of the PCR-dipstick DNA chromatography method was higher than that of traditional bacterial culture. Bacterial culture mainly isolates dominant bacteria, while other bacteria present at lower concentrations may be masked by the dominant ones. A total of 23 cases of *P. aeruginosa* were cultured, and in one case, the result was inconsistent with that of the PCR-dipstick DNA chromatography method. The PCR-dipstick DNA chromatography method detected a mixed infection of *P. aeruginosa* and *A. baumannii* (**Figure 5I**—126). It is presumed that the low concentration of *A. baumannii* in the sample was overshadowed by the dominant *P. aeruginosa* during cultivation. Additionally, 24 cases of *K. pneumoniae* were cultured, and in three cases, the results differed from those of the PCR-dipstick DNA chromatography method (**Figure 5I**—131, 133, 137). In these three cases, the PCR-dipstick DNA chromatography method indicated a mixed infection of *K. pneumoniae* and *M. pneumoniae*. The inconsistency arose because the culture conditions for *M. pneumoniae* were stringent, and conventional bacterial culture failed to cultivate it. Therefore, even if the bacterial concentration of *M. pneumoniae* was high in these samples, conventional culture methods may still miss it. A total of six cases of *H. influenzae* were cultured, and in two cases, the results were inconsistent with those of the PCR-dipstick DNA chromatography method (**Figure 5I**—123, 125). These two cases were detected as mixed infections by PCR-dipstick DNA chromatography, but the presence of other bacteria at low concentrations led to their overshadowing by the dominant *H. influenzae* during cultivation. Additionally, 12 cases of *S. pneumoniae* and 15 cases of *S. aureus* were cultured, and in one case, the result was inconsistent with that of the PCR-dipstick DNA chromatography method (**Figure 5I**—124). The PCR-dipstick DNA chromatography detected a mixed infection of *S. aureus* and *P. aeruginosa*, but the growth of *P. aeruginosa* was obscured by the dominant *S. aureus*, resulting in a false negative. In total, nine cases of mixed infections were identified via bacterial culture, and these samples also indicated mixed infections through PCR-dipstick DNA chromatography (**Figure 5I**—127, 128, 129, 130, 132, 134,135, 136, 139). However, bacterial cultures failed to detect *M. pneumoniae* in **Figure 5I** (132 and 135), while the low concentration of *A. baumannii* in **Figure 5I** (132) also went undetected. The results of the 17 cases of mixed-infection samples tested by the PCR-dipstick DNA chromatography assay and their corresponding bacterial culture results are presented in **Table 2**. Out of the 186 samples, 73 were negative for bacterial culture, primarily because the culturing conditions for *M. pneumoniae* were stringent and *C. pneumoniae* is an intracellular microorganism that cannot be cultured *in vitro*, making traditional culturing methods ineffective for detecting these pathogens.

**Table 2:** The results of 17 cases of mixed-infection samples tested by the PCR-dipstick DNA chromatography assay and their corresponding bacterial culture results.

Sample number	Multiplex PCR-dipstick DNA chromatography assay	Bacterial culture
123	*P. aeruginosa, H. influenzae, S. aureus*	*H. influenzae*
124	*S. aureus*, *P. aeruginosa*	*S. aureus*
125	*A. baumannii*, *P. aeruginosa*, *H. influenzae*	*H. influenzae*
126	*A. baumannii*, *P. aeruginosa*	*P. aeruginosa*
127	*A. baumannii*, *K. pneumoniae*, *H. influenzae*	*A. baumannii*, *K. pneumoniae*, *H. influenzae*
128	*A. baumannii*, *P. aeruginosa*	*A. baumannii*, *P. aeruginosa*
129	*K. pneumoniae*, *H. influenzae*	*K. pneumoniae*, *H. influenzae*
130	*A. baumannii*, *P. aeruginosa*, *H. influenzae*	*A. baumannii*, *P. aeruginosa*, *H. influenzae*
131	*K. pneumoniae*, *M. pneumoniae*	*K. pneumoniae*
132	*A. baumannii*, *P. aeruginosa*, *H. influenzae*, *S. aureus*, *M. pneumoniae*	*P. aeruginosa*, *H. influenzae*, *S. aureus*
133	*K. pneumoniae*, *M. pneumoniae*	*K. pneumoniae*
134	*A. baumannii*, *P. aeruginosa*, *H. influenzae*	*A. baumannii*, *P. aeruginosa*, *H. influenzae*
135	*P. aeruginosa*, *H. influenzae*, *S. aureus*, *M. pneumoniae*	*P. aeruginosa*, *H. influenzae*, *S. aureus*
136	*K. pneumoniae*, *H. influenzae*	*K. pneumoniae*, *H. influenzae*
137	*K. pneumoniae*, *M. pneumoniae*	*K. pneumoniae*
138	*A. baumannii*, *P. aeruginosa*, *K. pneumoniae*	*A. baumannii*
139	*H. influenzae*, *S. pneumoniae*, *S. aureus*	*H. influenzae*, *S. pneumoniae*, *S. aureus*

### DNA sequencing

In order to verify the accuracy of the PCR-dipstick chromatography method for detecting PCR products, the PCR products of all clinical samples were subjected to DNA sequencing. DNA sequencing results were completely consistent with the qualitative results of PCR-dipstick chromatography assay, which proves no false negative and false positive for target detection. Consequently, the consistency between PCR-dipstick chromatography assay and DNA sequencing was 100%, and the kappa value was 1.00.

## Discussion

In the present study, a PCR-dipstick chromatography assay capable of simultaneously detecting eight respiratory bacterial pathogens was successfully developed. The results demonstrated that all eight target bacteria could be precisely identified using PCR-dipstick chromatography, and no cross-reactivity was observed. The LOD of PCR-dipstick chromatography for individual bacteria ranges from 10 to 10^2^ CFU/mL. There was no cross-reaction among the eight bacteria, nor among representative respiratory bacteria such as *A. junii*, *E. cloacae*, *K. oxytoca*, *H. parainfluenzae*, *P. fluorescens*, *A. hydrophila*, and *S. epidermidis*, highlighting the excellent specificity of the PCR-dipstick DNA chromatography method. The concordance rate between PCR-dipstick chromatography and DNA sequencing was 100%, indicating that the PCR-dipstick chromatography assay is simple, rapid, high-throughput, sensitive, and accurate and exhibits good diagnostic performance.

One of the prominent advantages of the PCR-dipstick chromatography method lies in its capacity to detect mixed pathogens within a single sample. We applied this assay to evaluate 186 clinical sputum/BALF samples and identified 17 cases of multiple pathogen infections. In contrast, the traditional bacterial culture method, regarded as the gold standard for diagnosis, detected only nine cases of mixed infections. Moreover, for bacteria present at low concentrations, dominant species during culture can mask their detection, leading to false negatives. This not only hampers clinicians’ ability to prescribe appropriate antibiotics but also prolongs the duration of patient infections. Importantly, in recent years, infections caused by *M. pneumoniae* and *C. pneumoniae* have become increasingly prevalent in respiratory infections, accounting for approximately 10%–40% and 5%–10% of infections in children, respectively, with a 1%–2% co-infection rate of both pathogens (Chen et al., 2013; Defilippi et al., 2008; Hamano-Hasegawa et al., 2008). However, the cultivation conditions for *M. pneumoniae* are stringent and time-consuming and yield low positivity rates, rendering traditional bacterial culture inadequate for meeting the rapid diagnostic requirements of clinical practice (Zhao et al., 2020; Qu et al., 2022). As an intracellular microorganism, *C. pneumoniae* cannot be cultured *in vitro*. Consequently, traditional bacterial culture failed to detect *M. pneumoniae* and *C. pneumoniae* infections in the 186 samples analyzed. In our study, the PCR-dipstick chromatography method performed well in detecting infections caused by these two pathogens, with a sensitivity of 10 CFU/mL.

Quantifying the target pathogen is essential for differentiating the relative abundance of bacterial infections since changes in bacterial concentration before and after treatment can signify the effectiveness of the therapy. In the current study, when the bacterial concentration was between 10 and 10^7^ CFU/mL, the intensity of the color of the single-bacterial detection line increased with the rise in bacterial concentration (**Figure 4**). Therefore, we could approximately determine the change in single-bacterial concentration of the sample based on the color intensity of the DNA detection line for each order of magnitude. However, we noted that the relationship between the color intensity of the detection line and the bacterial concentration was weakened in the multi-target pathogen response. This could be attributed to the competition between streptavidin-coated blue latex microspheres. For bacteria of the same order, the detection line color of single bacteria was stronger than that of multiple target bacteria. The semi-quantitative results for multiple pathogens were lower than the actual bacterial concentration. Nevertheless, even though the semi-quantitative results of multiple infection samples were not accurate, as a qualitative analysis, the PCR-dipstick chromatography assay could still indicate which bacteria dominated in the same sample, which is of significant clinical value.

The most conspicuous limitation of the PCR-dipstick chromatography assay was the inaccurate quantification of multiple pathogens in a sample. Consequently, clinicians must make a comprehensive judgment based on the sample source, disease conditions, and the relative concentrations of multiple pathogens. To overcome this drawback, we will further investigate the relationship between the detection line color intensity of multiple infections and the concentration of bacteria by analyzing a large number of multiple infection clinical samples. Additionally, the PCR-dipstick chromatography method can only analyze a limited number of species, while the bacteria associated with respiratory infection are complex and diverse. A single strip could only identify eight related bacterial pathogens, which oversimplified the actual microflora of respiratory tract infection. Therefore, a negative sample identified by the PCR-dipstick chromatography method could only indicate the absence of the eight bacterial infections, but other pathogenic microorganisms could not be excluded. In fact, the defined bacterial species may not be directly involved in the etiology of a disease, and pathogens other than these eight bacteria may also be the main causative agents. Although the test strip DNA biosensor platform can be constructed according to different needs and used for multiple detections of different pathogens, with the continuous addition of targets in a strip, the competition between target primers and non-specific amplifications will increase, and sensitivity and specificity will decline, making it difficult to achieve ultra-high-throughput detection. With the development of the signal processing, probe, and multiplex PCR technologies of the strip biosensor, additional bacterial species related to respiratory tract infections should be incorporated into the PCR-dipstick chromatography method.

## Conclusion

A highly effective multiplex PCR-dipstick DNA chromatography assay was successfully established. This assay enabled the simultaneous detection of eight respiratory bacterial pathogens and was seamlessly integrated into clinical sample analysis. The results unequivocally suggest that this single-use, disposable device holds substantial promise for dissecting the microbial composition associated with respiratory infections. Its simplicity and portability make it an ideal choice for small laboratories and field diagnostics, potentially revolutionizing the way respiratory infections are diagnosed and managed.

## Data Availability

The original contributions presented in the study are included in the article/supplementary material. Further inquiries can be directed to the corresponding author.
